# Functional activity of peripheral blood eosinophils in allergen-induced late-phase airway inflammation in asthma patients

**DOI:** 10.1186/s12950-015-0065-4

**Published:** 2015-03-29

**Authors:** Simona Lavinskiene, Kestutis Malakauskas, Jolanta Jeroch, Deimante Hoppenot, Raimundas Sakalauskas

**Affiliations:** Department of Pulmonology and Immunology, Lithuanian University of Health Sciences, Kaunas, Lithuanian

**Keywords:** Eosinophils, Airway inflammation, Allergic asthma, Apoptosis, Chemotaxis, ROS

## Abstract

**Objective:**

We aimed to investigate peripheral blood eosinophil chemotaxis, generation of spontaneous reactive oxygen species (ROS), and apoptosis in patients with allergic asthma after bronchial allergen challenge.

**Material and methods:**

A total of 18 patients with allergic asthma (AA), 14 with allergic rhinitis (AR), and 10 healthy subjects (HS) underwent bronchial challenge with a specific allergen extract. Eosinophils from peripheral blood were isolated 24 h before as well as 7 and 24 h after bronchial allergen challenge. Chemotaxis, spontaneous ROS production in eosinophils, and apoptosis were analyzed by flow cytometry. Serum and induced sputum IL-5 levels were measured by ELISA; the cell count in sputum was analyzed by the May-Grünwald-Giemsa method.

**Results:**

Before bronchial allergen challenge, peripheral blood eosinophil chemotaxis, spontaneous ROS production was enhanced and eosinophil apoptosis was reduced in the patients with AA as compared with AR patients and HS (*P* < 0.05). Meanwhile, eosinophil chemotaxis and ROS generation markedly increased in the patients with AA 7 h and 24 h after challenge compared with other groups and baseline values (*P* < 0.05). The percentage of apoptotic eosinophils in the patients with AA decreased at 7 h as well as 24 h after challenge when compared with other groups and the baseline values (*P* < 0.05). There was a significant correlation between the migrated peripheral blood eosinophil count and the sputum eosinophil count (*Rs* = 0.89, *P* < 0.0001) and the sputum IL-5 level (*Rs* = 0.68, *P* = 0.002) at 24 h after bronchial challenge only in the patients with AA. Furthermore, the percentage of peripheral blood apoptotic eosinophils significantly correlated with eosinophil count in sputum (*Rs* = −0.53, *P* = 0.02), and ROS production correlated with the serum IL-5 levels (*Rs* = 0.71, *P* = 0.01).

**Conclusion:**

During allergen-induced late-phase airway inflammation, peripheral blood eosinophils demonstrated further alterations of their functional activity manifested by enhanced spontaneous ROS production, increased chemotaxis, and diminished apoptosis in patients with AA.

## Introduction

Asthma is an inflammatory disorder of the airways involving T‐cells, mast cells, and eosinophils [1]. The toxic components of eosinophils are thought to be important in inducing bronchial mucosal injury and dysfunction [2]. Following airway allergen exposure, the development of airway eosinophilia is associated with increased IL-5 expression in the sputum, elevated concentrations of IL-5 in luminal fluid and serum, and a heightened capacity of airway cells for ex vivo generation of IL-5 [3-5].

IL-5 plays a key role in eosinophil proliferation, differentiation, maturation, migration to tissue sites and survival, as well as prevention of eosinophil apoptosis [6,7]. Eosinophil chemotaxis to the lungs during allergic airway inflammation represents a major part of the inflammation process [8]. Transmigration of the eosinophil through the vascular endothelium is a multistep process; rolling, tethering, firm adhesion, and transendothelial migration are regulated by the coordinated interaction between networks involving chemokine, cytokine and adhesion molecules [9,10]. Experiments with in vitro allergen as well as endobronchial allergen challenge have shown that blood and bronchoalveolar lavage eosinophils from subjects with asthma have a greater responsiveness to chemoattractants and enhanced chemotaxis [11,12]. During the process of allergic inflammation, eosinophils release not only toxic granule proteins but also reactive oxygen species (ROS), which are known to cause tissue damage [13]. It has been demonstrated that allergic patients have upregulated oxidative metabolism in blood eosinophils when compared with healthy subjects [14,15]. It leads to the observations that eosinophils isolated from allergic patients might be already activated in peripheral blood streams before they infiltrate the tissue.

In the absence of any inflammatory survival-prolonging factors, eosinophils die by apoptosis in a few days, but in inflamed airways, eosinophils survival is thought to be prolonged due to the surrounding proinflammatory factors such as IL-5, IL-3, and granulocyte-macrophage colony-stimulating factor [16,17]. There are some data about impaired peripheral blood eosinophil apoptosis in allergic patients [18], and this might contribute to greater airway eosinophilia.

There is no doubt that eosinophils are important cells which participate in allergic airway inflammation. Meanwhile, associations between eosinophil infiltration in the airways and peripheral blood eosinophil chemotaxis, ROS production, and apoptosis have not been completely elucidated yet. Therefore, the regulation of blood eosinophil activity in asthmatic patients especially after allergen challenge needs to be investigated.

We hypothesized that evaluating peripheral blood eosinophils functional activity (chemotaxis, apoptosis, and ROS production) during allergen-induced late-phase airway inflammation is important for understanding the pathogenesis of eosinophilic inflammation in the airways.

## Methods

### Subjects

A total of 42 nonsmoking adults (13 men and 29 women) were examined: 18 patients with intermittent or mild-to-moderate persistent allergic asthma, defined according to the GINA criteria [19], 14 patients with mild-to-moderate persistent allergic rhinitis, defined according to the ARIA criteria [20], and 10 healthy subjects who comprised the control group. The patients were recruited from the Department of Pulmonology and Immunology, Hospital of the Lithuanian University of Health Sciences, Kaunas. The study protocol was approved by the Regional Biomedical Research Ethics Committee of the Lithuanian University of Health Sciences (BE-2-23), and each participant gave his/her informed written consent. The study was registered in the U.S. National Institutes of Health trial registry *ClinicalTrials.gov* with identifier NCT02214303.

Patients with allergic asthma and rhinitis had a clinical history of the disease for ≥1 year, current symptoms, and positive results of skin prick test (≥3 mm) with the following allergens: *Dermatophagoides pteronyssinus* (*D. pteronyssinus*), birch pollen, or mixture of 5 grasses. All the patients were not using inhaled, nasal, or oral steroids at least 1 month before visits; short-acting *β*2 agonists, at least 12 h; long-acting *β*2 agonists, at least 48 h prior the lung function test, and antihistamines and antileukotrienes, 7 days before the skin prick test and the lung function test. None of the patients had a history of smoking. Baseline forced expiratory volume in one second (FEV_1_) was more than 70% of the predicted value in all patients. All the healthy subjects were nonsmokers, without symptoms of asthma or rhinitis, with normal findings of spirometry, and all showed negative results of the skin prick test.

### Skin prick and lung function testing

All the patients were screened for allergy by the skin prick test using standardized allergen extracts (Stallergenes S.A., France) for the following allergens: *D. pteronyssinus*, *D. farinae*, cat and dog dander, mixture of pollen of 5 grasses, birch pollen, mugwort, *Alternaria*, *Aspergillus*, and *Cladosporium*. Histamine hydrochloride (10 mg/mL) was used for a positive control. Skin testing was read 15 min after application. The results of the skin prick test were considered positive if the mean wheal diameter was ≥ 3 mm [21].

Pulmonary function was tested using a pneumotachometric spirometer “CustovitM” (Custo Med, Germany). Baseline FEV_1_, forced vital capacity (FVC), and FEV_1_/FVC ratio were recorded as the highest of three reproducible measurements. The results were compared with the predicted values matched for age, body height, and sex according to the standard methodology [22].

### Measurement of airway responsiveness to methacholine

Airway responsiveness was assessed as changes in the airway function after challenge with inhaled methacholine using a reservoir method [23]. Methacholine was nebulized into a 10-L reservoir with a pressure nebulizer (Pari Provocation I; Pari, Stanberg, Germany). Aerolized methacholine was inhaled through a one-way valve at 5-min intervals starting with 15-*μ*g methacholine dose and doubling it until a 20% decrease in FEV_1_ from the baseline or the total cumulative dose of 3.87 mg was achieved. The bronchoconstricting effect of each dose of methacholine was expressed as a percentage of decrease in FEV_1_ from the baseline value. The provocative dose of methacholine causing a ≥ 20% fall in FEV_1_ (PD_20_) was calculated from the log dose–response curve by linear interpolation of two adjacent data points.

### Peripheral blood collection and isolation of eosinophils

Peripheral blood samples for eosinophil isolation were collected into sterile vacutainers with ethylenediaminetetraacetic acid (EDTA). Polymorphonuclear leukocytes (PMNs) were isolated by high density gradient centrifugation. The whole blood was layered on Ficoll-Paque PLUS (GE Healthcare, Finland) and centrifuged at 1000 *g* for 30 min at room temperature. PMNs were separated by hypotonic lysis of erythrocytes and eosinophils were separated using a magnetic eosinophil isolation kit (Miltenyi Biotek, USA). Isolated eosinophils were diluted in cell culture RPMI 1640 media (Biological Industries, Israel) at a final concentration of 2 × 10^6^/mL. The viability of eosinophil was checked flow cytometrically using propidium iodide (2 mg/mL) and it always was > 95 %.

### Sputum induction and processing

The subjects inhaled 10 mL of sterile hypertonic saline solution (3%, 4%, or 5% NaCl, Ivex Pharmaceuticals, USA) at room temperature from an ultrasonic nebulizer (DeVilbiss Health Care, USA). The duration of each inhalation was 7 min, and it was stopped after expectoration an adequate amount of sputum. In order to detect a possible decrease in FEV_1_, spirometry was performed after each inhalation. Sputum was poured into a Petri dish and separated from saliva. A 4-fold volume of freshly prepared 0.1% dithiothreitol (DTT; Sigma-Aldrich) was added. The mixture was vortexed and placed on a bench rocker for 15 min at room temperature. Next, an equal volume of phosphate-buffered saline solution (PBS; Sigma-Aldrich) was added to DTT. The cell pellet was separated using a 40-*μ*m cell stainer (Becton Dickinson, USA). The mixture was centrifuged for 10 min at 4°C; the supernatant was aspirated and stored at −70°C for later analysis. The total cell counts, percentage of epithelial cells, and cell viability were investigated using a Neubauer hemocytometer (Heinz-Herenz, Germany) under a microscope (B5 Professional, Motic, China) by employing the Trypan blue exclusion method. The cytospin samples of induced sputum were prepared using a cytofuge instrument (Shandon Southern Instruments, USA).

### Induced sputum cell analysis

The prepared sputum cytospins were stained by the May-Grünwald-Giemsa method for differential cell counts. Cell differentiation was determined by counting approximately 400 cells in random fields of view under a light microscope, excluding squamous epithelial cells. The cells were identified by standard morphological criteria, nuclear morphology, and cytoplasmic granulation. Cell counts were expressed as percentages of total cells and absolute values (10^6^/L).

## Peripheral blood eosinophil chemotaxis, apoptosis and ROS production assay

### Chemotaxis in vitro

Eosinophil chemotaxis in vitro was performed in a 10-well cell transmigration chamber (Neuro Probe, USA). The lower and upper wells of chamber were isolated by a polyvinylpyrrolidone (PVP)-treated polycarbonate track-etch membrane, containing 2 × 10^6^ 3 *μ*m/mm^2^ pores (Neuro Probe, USA). The lower wells were pre-filled with isotonic Percoll (GE Healthcare, Finland) and eotaxin, a chemotactic factor, at different concentrations (10, 100, or 1000 ng/mL). RPMI 1640 was used as a negative control. The upper wells were filled with eosinophil culture suspension (1 × 10^3^/mL) and incubated for 2 h (37°C, 5% CO_2_).

After the incubation, the suspensions of upper and lower wells were resuspended in tubes for flow cytometry. Nonmigrated eosinophils remained in the upper wells. The migration rate was calculated from the total number of eosinophils harvested from the lower well and expressed as percentage of the total input of eosinophils into the upper well.

The number of migrated eosinophils was calculated by flow cytometry using Liquid Counting Beads (BD Biosciences, USA) according to the manufacturer’s recommendations. The amount of migrated eosinophils was expressed in percentages.

### Apoptosis

Isolated eosinophils were resuspended in the annexin-binding buffer (pH 7.4) containing 50 mM HEPES, 700 mM NaCl, 12.5 mM CaCl2 (Invitrogen, USA) and incubated with fluorescein isothiocyanate-labeled (FITC)-annexin V (Invitrogen, USA) and propidium iodide (PI) for 15 min at room temperature in the dark. After the incubation, apoptosis was analyzed by flow cytometry using the CellQuest software (BD Biosciences, USA). Apoptotic cells were quantified as the percentage of the total population that was positive for FITC, but negative for PI. Necrotic cells were positive for PI.

### Analysis of ROS production

Spontaneous ROS production in peripheral blood eosinophils was performed in sterile 96-well microplates (Falcon, BD, USA). For the detection of generated ROS, dihydrorhodamine-123 (DHR-123, 750 ng/mL final, Invitrogen, USA), a nonfluorescent dye, was added. DHR-123, interacting with intracellular ROS, is oxidized to the green-fluorescent rhodamine-123.The plates were filled with eosinophil cultures and incubated for 45 min (37°C, 5% CO_2_). The relative amount of generated ROS was measured flow cytometrically by determination of mean green fluorescence intensity in the eosinophil population.

### Detection of cytokine in serum and induced sputum supernatant

The serum and sputum IL-5 levels were measured by an enzyme-linked immunosorbent assay (ELISA) according to the manufacturer’s instructions (Abcam, USA). The minimum detectable concentration was 5 pg/mL.

The peripheral blood cell analysis was performed on an automated hematology analyzer (Sysmex XE-5000, Japan).

### Statistical analysis

Statistical analysis was performed by using the Statistical Package for Social Sciences, version 17.0 for Windows (SPSS 17.0). The normality assumption of data was verified with the Kolmogorov-Smirnov test. The data were expressed as a median and a range. The results of methacholine PD_20_ measurements are expressed as a geometric mean. PD_20_ values were log-transformed for analysis to fit a normal distribution.

Due to a skewed distribution of the variable, nonparametric tests were applied. The Kruskal-Wallis test was used to evaluate differences between the groups of patients and the control group. Differences between 2 independent groups were determined by the Mann–Whitney *U* test. Differences among 3 and more paired samples were evaluated by the Friedman test*.* Differences between 2 dependent samples were evaluated by the Wilcoxon test. The Spearmen rank test was used to assess relationships between measurements. Statistical significance was assumed at a *P* value of <0.05.

## Results

### Characteristics of studied subjects

A total of 42 nonsmoking adults (17 men and 25 women; mean age 31 ± 9 years) were examined: 18 patients with intermittent or mild-to-moderate persistent allergic asthma, 14 patients with mild-to-moderate persistent allergic rhinitis, and 10 healthy subjects who comprised the control group. There were no significant age and gender differences comparing the groups. Twenty-one patients were sensitized to *D. pteronyssinus*; 5 patients, to birch pollen; and 6 patients, to a mixture of pollen of 5 grasses. The mean wheal diameter induced by an allergen was similar in both groups of patients. The demographic and clinical data of the study subjects are presented in Table 1. There were no significant difference in the baseline FEV_1_ (% of predicted) the comparing all groups. A provocative dose of methacholine causing a 20% decrease in FEV_1_ (PD_20_) was documented in 18 patients with allergic asthma and 1 patient with allergic rhinitis.

**Table 1:** **Demographic and clinical characteristics of study subjects**

**Characteristics**	**Patients with allergic asthma N = 18**	**Patients with allergic rhinitis N = 14**	**Healthy subjects N = 10**
Age (years), median (range)	31 (21–50)	30 (18–49)	28 (22–45)
Sex (male/female), n	10/8	4/10	3/7
Wheal diameter induced by allergen (mm), median (range)	6.2 (4–11)	7.8 ± 1.8 (4–13)	0
Sensitization to *D. pteronyssinus*/birch/5 grass mixture allergen, n	13/3/2	9/3/2	0
PD_20_ (mg), geometric mean (range)	0.38 (0.25–0.54)	0.52*	0/0/0
FEV_1_ (% of predicted), mean ± SD	98 ± 15	105 ± 10	102 ± 11

*N = 1 because methacholine challenge provoked bronchoconstriction only to one allergic rhinitis patient.

PD_20_ - a provocative dose of methacholine causing a 20% decrease in FEV_1_;

FEV_1_ - forced expiratory volume in one second.

### Eosinophil composition of peripheral blood and induced sputum

**Figure 1:**
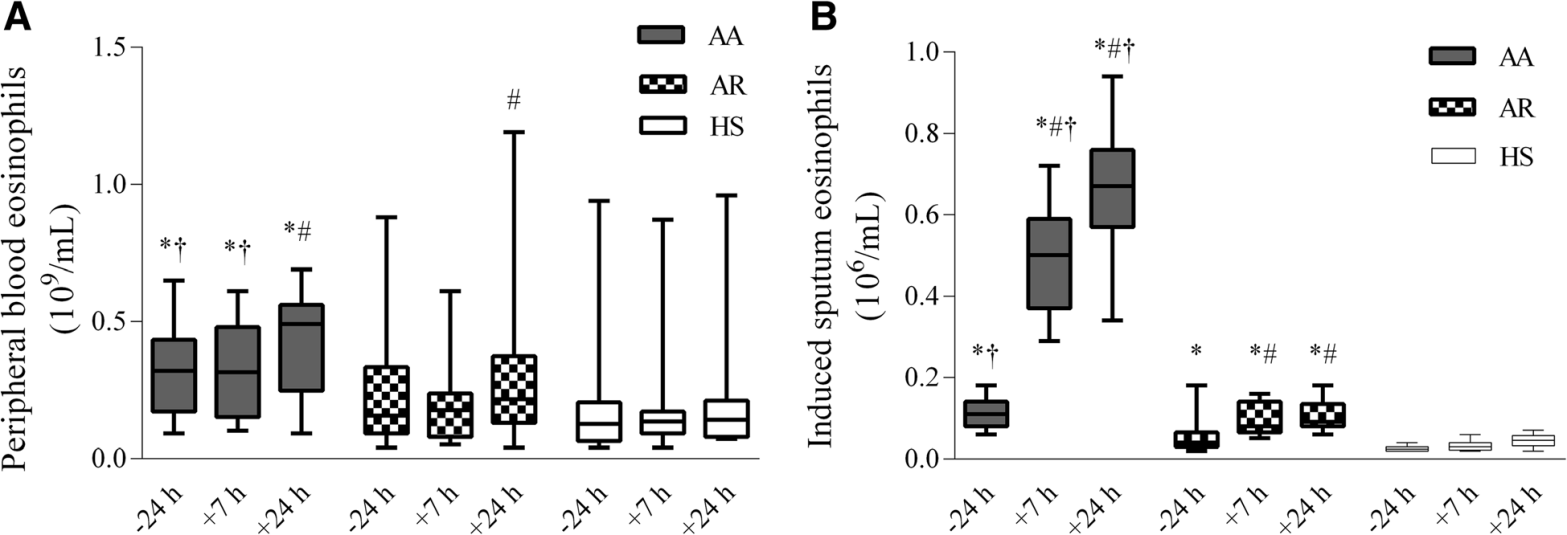
**Eosinophil count of patients with allergic asthma, allergic rhinitis and healthy subjects. A** Eosinophil counts in peripheral blood 24 h before as well as 7 h and 24 h after bronchial challenge. **B** Eosinophil counts in the induced sputum 24 h before as well as 7 h and 24 h after bronchial challenge. Data are shown as median (range). AA indicates patients with allergic asthma (n = 18); AR, patients with allergic rhinitis (n = 14); HS, healthy subjects (n = 10). *P < 0.05 compared with healthy subjects; #*P* < 0.05 compared with baseline values; †*P* < 0.05 compared with patients with allergic rhinitis.

The eosinophil count in the peripheral blood 24 h before bronchial allergen challenge was significantly higher in the patients with allergic asthma compared with the patients with allergic rhinitis and healthy subjects (0.32 × 10^9^/L [range, 0.09–0.65] vs. 0.16 × 10^9^/L [0.04–0.88] and 0.13 × 10^9^/L [0.04–0.94], *P* <0.05). At 24 hours after bronchial challenge, the peripheral blood eosinophil count was significantly increased in the patients with allergic asthma compared with the baseline values and the healthy subjects (Figure 1A). The increased peripheral blood eosinophil count also was recorded 24 h after bronchial challenge in the patients with allergic rhinitis compared with the baseline values (0.22 × 10^9^/L [range, 0.04–1.19] vs. 0.16 × 10^9^/L [range, 0.04–0.88], *P* < 0.05). Meanwhile, at the baseline as well as 7 h and 24 h after bronchial challenge, the total eosinophil count in the sputum was significantly higher in the patients with allergic asthma than those with allergic rhinitis and the healthy subjects (*P* < 0.05) (Figure 1B). At the baseline as well as 7 h and 24 h after bronchial challenge, the patients with allergic rhinitis showed a significantly greater eosinophil count in the sputum than the healthy subjects (*P* < 0.05). Bronchial allergen challenge had no impact on eosinophil count in healthy subjects.

## Functional activity of peripheral blood eosinophils

### Peripheral blood eosinophil chemotaxis in vitro

**Figure 2:**
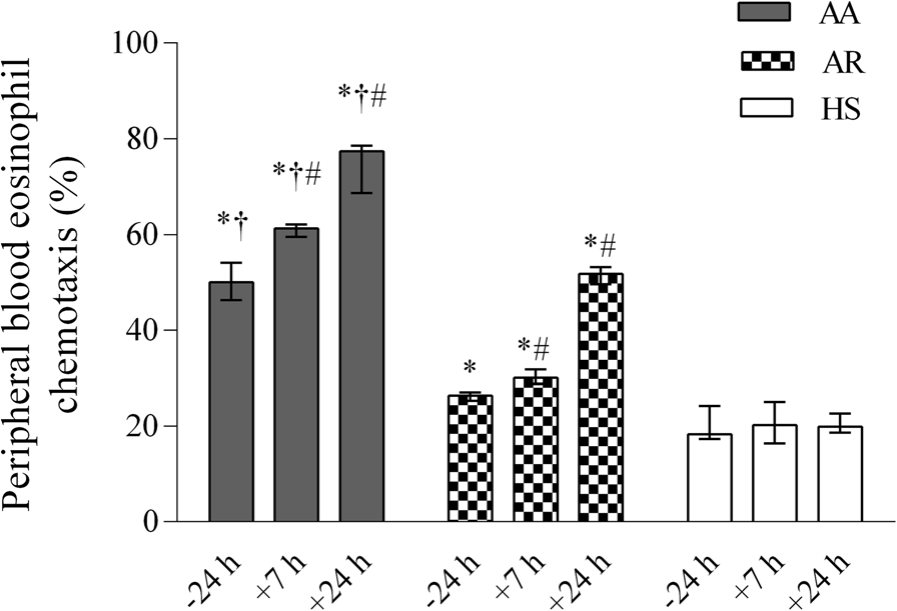
**Peripheral blood eosinophil chemotaxis (stimulated with 1000 ng/mL of eotaxin) in patients with allergic asthma, patients with allergic rhinitis, and healthy subjects 24 h before as well as 7 h and 24 h after bronchial challenge.** Data are shown as median (range). AA indicates patients with allergic asthma (n = 18); AR, patients with allergic rhinitis (n = 14); HS, healthy subjects (n = 10). *P < 0.05 compared with healthy subjects; #*P* < 0.05 compared with baseline values; †*P* < 0.05 compared with patients with allergic rhinitis.

Eotaxin at different concentrations (10, 100, and 1000 ng/mL) had an impact on peripheral blood eosinophil chemotaxis in all the studied groups, but the highest concentration had the greatest effect. At the baseline, peripheral blood eosinophil chemotaxis after the stimulation with 1000 ng/mL of eotaxin was higher in the patients with allergic asthma compared with those with allergic rhinitis and the healthy subjects (*P* < 0.05). At 7 h and 24 h after bronchial challenge, eosinophil chemotaxis was significantly enhanced in the patients with allergic asthma compared with the baseline values, and it was greater than in the patients with allergic rhinitis and the healthy subjects (Figure 2). Meanwhile, bronchial allergen challenge had no significant effect on eosinophil chemotaxis in the peripheral blood of healthy subjects.

### ROS in peripheral blood eosinophils

**Figure 3:**
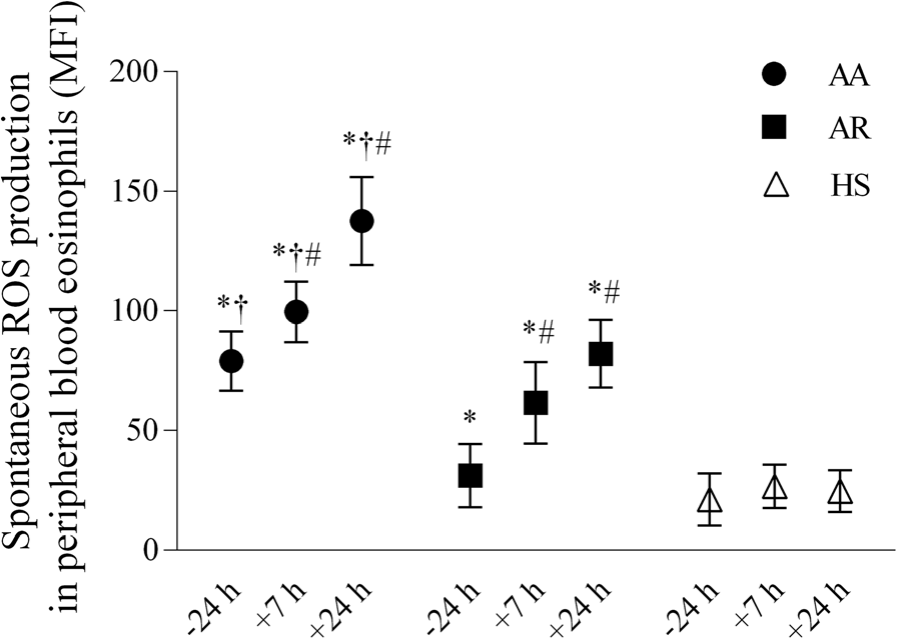
**Production of reactive oxygen species in peripheral blood eosinophils of patients with allergic asthma, patients with allergic rhinitis, and healthy subjects 24 h before as well as 7 h and 24 h after bronchial challenge.** Data are shown as median (range). AA indicates patients with allergic asthma (n = 18); AR, patients with allergic rhinitis (n = 14); HS, healthy subjects (n = 10); MFI, mean fluorescence intensity. *P < 0.05 compared with healthy subjects; #*P* < 0.05 compared with baseline values; †*P* < 0.05 compared with patients with allergic rhinitis.

Before bronchial allergen challenge, spontaneous ROS production in peripheral blood eosinophils was significantly greater in the patients with allergic asthma compared than those with allergic rhinitis and the healthy subjects (*P* < 0.05). At 7 h and 24 h after bronchial challenge, ROS generation was significantly greater in the patients with allergic asthma compared with other groups and the baseline values (Figure 3). Bronchial allergen challenge had no impact on ROS production in eosinophils isolated from the healthy subjects.

### Apoptosis of peripheral blood eosinophils

**Figure 4:**
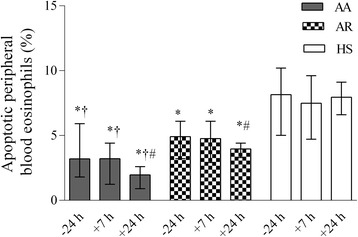
**The percentage of apoptotic peripheral blood eosinophils in patients with allergic asthma, patients with allergic rhinitis, and healthy subjects 24 h before as well as 7 h and 24 h after bronchial challenge.** Data are shown as median (range). AA indicates patients with allergic asthma (n = 18); AR, patients with allergic rhinitis (n = 14); HS, healthy subjects (n = 10). *P < 0.05 compared with healthy subjects; #*P* < 0.05 compared with baseline values; †*P* < 0.05 compared with patients with allergic rhinitis.

Before bronchial allergen challenge, the patients with allergic asthma had a significantly lower percentage of apoptotic peripheral blood eosinophils than those with allergic rhinitis and the healthy subjects (*P* < 0.05) (Figure 4). Furthermore, 7 h and 24 h after bronchial challenge, a significantly lower percentage of apoptotic peripheral blood eosinophils was recorded in the asthma patents’ group when compared with other groups and the baseline values (*P* < 0.05).

### IL-5 Levels in induced sputum and serum

**Figure 5:**
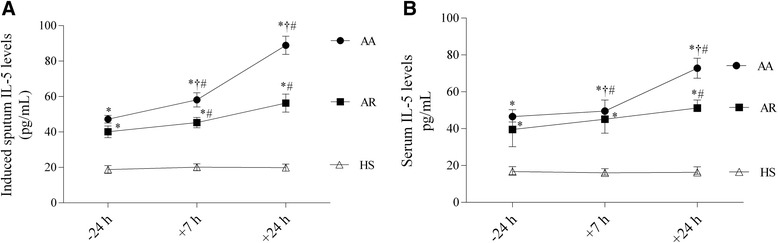
**IL-5 levels of patients with allergic asthma, patients with allergic rhinitis, and healthy subjects. A** IL-5 levels in the induced sputum 24 h before as well as 7 h and 24 h after bronchial allergen challenge. **B** IL-5 levels in serum 24 h before as well as 7 h and 24 h after bronchial allergen challenge. Data are shown as median (range). AA indicates patients with allergic asthma (n = 18); AR, patients with allergic rhinitis (n = 14); HS, healthy subjects (n = 10). *P < 0.05 compared with healthy subjects; #*P* < 0.05 compared with baseline values; †*P* < 0.05 compared with patients with allergic rhinitis.

There was a significant increase in the induced sputum IL-5 levels in the patients with allergic asthma and those with allergic rhinitis compared with the healthy subjects at 24 h before bronchial challenge (Figure 5A). At 7 h and 24 h after bronchial challenge, the induced sputum IL-5 levels increased significantly in the patients with allergic asthma and those with allergic rhinitis compared with the healthy subjects. Moreover, the sputum IL-5 levels at 7 h and 24 h after bronchial challenge were significantly higher in the patients with allergic asthma than those with allergic rhinitis. The same tendency was observed while analyzing serum IL-5 levels (Figure 5B). However, in patients with allergic rhinitis, there was no significant difference in the serum IL-5 levels comparing the baseline values with those recorded 7 h after bronchial challenge. Bronchial challenge had no impact on the IL-5 levels in the sputum and serum of healthy subjects.

### Correlations

**Figure 6:**
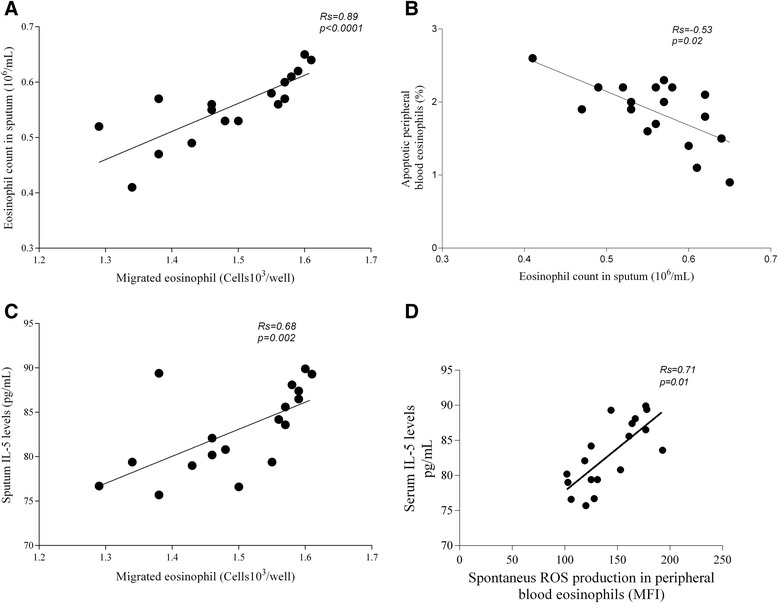
**Correlations between peripheral blood eosinophil activity and eosinophil count as well as IL-5 levels in the induced sputum in the patients with allergic asthma 24 h after bronchial allergen challenge** ***.*** **A** Correlation between migrated peripheral blood eosinophil count and eosinophil count in sputum. **B** Correlation between percentage of apoptotic peripheral blood eosinophils and eosinophil count in sputum. **C** Correlation between migrated peripheral blood eosinophil count and IL-5 level in sputum. **D** Correlation between ROS production in peripheral blood eosinophils and serum IL-5 levels. MFI indicates mean fluorescence intensity.

Significant correlations were found only in the patients with allergic asthma at 24 h after bronchial allergen challenge. The migrated peripheral blood eosinophil count significantly correlated with the eosinophil count in the sputum (*R*s = 0.89, *P* < 0.0001; Figure 6A). Moreover, there was a significant correlation between the percentage of apoptotic peripheral blood eosinophils and eosinophil count in sputum (*R*s = −0.53, *P* =0.02; Figure 6B). The migrated peripheral blood eosinophil count significantly correlated with sputum IL-5 levels (*R*s = 0.68, *P* = 0.002; Figure 6C), and ROS production in peripheral blood eosinophils significantly correlated with the serum IL-5 levels (*R*s = 0.71, *P* = 0.01; Figure 6D).

## Discussion

This study has demonstrated that peripheral blood eosinophil chemotaxis, spontaneous ROS production, and eosinophil apoptosis are altered in patients with allergic asthma during allergen-induced late-phase airway inflammation. The increased sputum eosinophil count and enhanced levels of IL-5 significantly correlated with the impaired functional activity of peripheral blood eosinophils.

It is known that eosinophils are recruited to sites of inflammation by released chemotactic agents. Eotaxin, a CC chemokine, is one of the strongest stimulator of eosinophil chemotaxis [24]. It also induces the release of various mediators from eosinophils and is known to play an integral role in the development of eosinophilic inflammation [25]. Therefore, in order to investigate chemotaxis *in vitro*, peripheral blood eosinophils were stimulated with different concentrations (10, 100, and 1000 ng/mL) of eotaxin. Our results showed that before bronchial allergen challenge, peripheral blood eosinophil chemotaxis stimulated with the highest eotaxin concentration was greatest in the patients with allergic asthma as compared with other groups. These data suggest that eosinophils in the peripheral blood of individuals with allergic asthma are already primed and more sensitive to a chemokine.

Experiments with *in vivo* injection of eotaxin into the skin of mice and rhesus monkeys showed local accumulation of eosinophils, and the kinetics of allergen-induced production of eotaxin is paralleled by eosinophil accumulation in a guinea-pig model of allergic airways [25,26].

We found that 7 h and 24 h after bronchial allergen challenge, eosinophil chemotaxis was significantly greater in the patients with allergic asthma than other groups and also compared with the baseline values. This shows that not only allergen challenge stimulates the functional activity of eosinophils, but response to eotaxin confirms the undeniable importance of this chemokine. Moreover, at 24 h after bronchial challenge, the increased eosinophil count in the sputum significantly correlated with the increased migrated eosinophil count in the peripheral blood of asthmatic patients. That can reflect the hallmark characteristic of allergic asthma by infiltration of eosinophils to the airway.

The inflammatory cells recruited to the asthmatic airways are exceptionally capable of producing ROS, resulting in abnormal physiologic function of DNA, proteins, and lipids that clinically can augment bronchial hyperresponsiveness and inflammation [27]. However due to the possibility that eosinophils can be already activated in periphery, we examined spontaneous ROS production in peripheral blood eosinophils.

Our study showed that spontaneous ROS production in peripheral blood eosinophils was significantly greater in the patients with allergic asthma than those with allergic rhinitis and the healthy subjects. Moreover, at 7 h and 24 h after bronchial allergen challenge, ROS generation in peripheral blood eosinophils was significantly greater in the patients with allergic asthma compared with the baseline values and other groups.

The upregulation of ROS production by blood eosinophils in allergic patients has been previously documented in several studies [14,28], and the results of these studies as well as our date suggest that eosinophils from allergic asthma patients may might be already activated in the peripheral blood stream before they infiltrate the tissues and the inhalation of aggravating compounds such as allergens can promote stronger activation of these cells.

Moreover, it was demonstrated that a low concentration of IL-5 enhanced chemokine-primed ROS production by eosinophils [14]. Consequently, we evaluated the levels of serum IL-5 and looked for a relationship with generated ROS in peripheral blood eosinophils. We found elevated serum IL-5 levels, especially 24 h after bronchial challenge, in the patients with allergic asthma compared with the baseline values and other groups. Moreover, a significant correlation was observed only 24 h after bronchial challenge and only in the patients with allergic asthma. The increased serum IL-5 levels correlated with the enhanced ROS production in peripheral blood eosinophils (*P* < 0.05). These findings suggest that enhanced ROS generation can activate a number of redox-sensitive signaling cascades, stimulate production-interaction of proinflammatory cytokines, and promote inflammation [29].

As it has been reported that IL-5 can enhance eosinophil migration by the upregulation of adhesion molecules on eosinophils [30], we analyzed sputum IL-5 levels and investigated the possible relationship to peripheral blood eosinophil chemotaxis. Thus, we determined a significant increase in sputum IL-5 levels in the patients with allergic asthma, and it correlated with the migrated peripheral blood eosinophil count in allergen-induced late-phase airway inflammation in asthma patients.

The accumulation of eosinophils in the asthmatic lungs is a complex process, which involves their maturation in and release from the bone marrow, adhesion and transmigration through the post-capillary endothelium, and then their chemotaxis to and activation/degranulation at inflammatory foci [9,31]. In the circulation and tissues, eosinophils are programmed to undergo apoptosis in the absence of viability-enhancing stimuli [31]. A defect in apoptosis might contribute to chronic tissue eosinophilia associated with asthma. Therefore, we aimed at evaluating peripheral blood eosinophil apoptosis during allergen-induced late-phase inflammation. Before bronchial challenge, the percentage of apoptotic peripheral blood eosinophils in the patients with allergic asthma was significantly lower compared with the patients with allergic rhinitis and the healthy subjects. Apoptosis of peripheral blood eosinophils in the patients with allergic asthma was more delayed 7 h and 24 h after bronchial challenge compared with the baseline and other groups. The same tendency was reported by Evans et al., who showed that in asthmatic patients demonstrating a late response, the survival of peripheral blood eosinophils is prolonged after the whole lung allergen challenge [32]. Moreover, Druilhe et al. and Vignola et al. demonstrated that asthmatic patients had greater numbers of eosinophils in the bronchial biopsy than healthy individuals [33,34]. All the above-mentioned results and findings of our study show that eosinophil survival through the inhibition of apoptosis can increase airway eosinophilia.

Finally, we evaluated relationships between eosinophil counts in the sputum and eosinophil viability after bronchi al allergen challenge, which could represent a link between eosinophilic influx to the airways possibly caused by delayed peripheral blood eosinophil apoptosis in allergen-induced late-phase airway inflammation.

Significant correlations were observed only 24 h after bronchial challenge and only in the patients with allergic asthma. The lower percentage of peripheral blood eosinophils was negatively correlated with the increased eosinophil count in the sputum. It might be that diminished apoptotic potential represents a mechanism that promotes resolution of eosinophilic inflammation in asthma.

In conclusion, this study has shown that allergic asthma patients demonstrates enhanced spontaneous ROS production, increased chemotaxis, and diminished apoptosis of peripheral blood eosinophils, and these alterations are more pronounced during allergen-induced late-phase airway inflammation.
